# Switchable Gene Expression in *Escherichia coli* Using a Miniaturized Photobioreactor

**DOI:** 10.1371/journal.pone.0052382

**Published:** 2013-01-17

**Authors:** Jae Myung Lee, Junhyeong Lee, Taesung Kim, Sung Kuk Lee

**Affiliations:** 1 School of Nano-Bioscience and Chemical Engineering, Ulsan National Institute of Science and Technology (UNIST), Ulsan, Republic of Korea; 2 School of Mechanical and Advanced Materials Engineering, UNIST, Ulsan, Republic of Korea; 3 School of Urban and Environmental Engineering, UNIST, Ulsan, Republic of Korea; Soonchunhyang University, Republic of Korea

## Abstract

We present a light-switchable gene expression system for both inducible and switchable control of gene expression at a single cell level in *Escherichia coli* using a previously constructed light-sensing system. The λ *c*I repressor gene with an LVA degradation tag was expressed under the control of the *ompC* promoter on the chromosome. The green fluorescent protein (GFP) gene fused to a λ repressor-repressible promoter was used as a reporter. This light-switchable system allows rapid and reversible induction or repression of expression of the target gene at any desired time. This system also ensures homogenous expression across the entire cell population. We also report the design of a miniaturized photobioreactor to be used in combination with the light-switchable gene expression system. The miniaturized photobioreactor helps to reduce unintended induction of the light receptor due to environmental disturbances and allows precise control over the duration of induction. This system would be a good tool for switchable, homogenous, strong, and highly regulatable expression of target genes over a wide range of induction times. Hence, it could be applied to study gene function, optimize metabolic pathways, and control biological systems both spatially and temporally.

## Introduction

Switchable gene expression systems would aid in reversible induction or repression of target genes at any desired time and hence reduce pleiotropic effects caused by over-expression. Precise and temporally switchable control of bacterial gene expression in response to external stimuli is an essential tool for understanding and manipulating complex biological systems. Efficient control of biological systems would, in turn, help achieve maximal production of the desired products by circumventing the problem of metabolic burden [1]–[3]. The inducible promoters commonly used for gene expression require the addition of a chemical (such as isopropyl β-d-1-thiogalactopyranoside (IPTG), anhydrotetracycline (aTC), or arabinose) or a change in physicochemical factors (such as pH, temperature, or ultraviolet (UV) light) [4]. Several switchable gene expression systems have been developed based on physicochemical stimuli [5], [6].

Chemical inducers such as aTC, arabinose, and IPTG are expensive and toxic; their separation from the final products requires additional downstream operations [7]. Moreover, it is difficult to temporarily halt or completely cease gene expression induced by a chemically regulated promoter because the chemical inducers are not readily removed. The use of thermo-regulatable promoters that can be induced by altering the temperature can cause protein aggregation, resulting in low yield of soluble proteins. Further, this is not a cost-effective switchable induction method, as rapid shifts in temperature are difficult to achieve [8], [9]. In the case of pH-inducible expression systems, changes in pH can affect both the structure and biochemical reactivity of cellular molecules due to an alteration in the optimal physiological pH [10]. For UV-inducible expression systems, the cells should be irradiated with UV to remove the caging group from its associated compound in order to restore its biological activity [11]. Weak irradiation cannot efficiently remove caging groups, but irradiation at higher intensity causes phototoxicity to the cells. Many of the above-mentioned disadvantages can be overcome by using visible light-inducible expression systems. Therefore, development of light-mediated gene expression systems is a rapidly advancing research field in the areas of functional genomics, synthetic biology, and biotechnology [1], [12], [13].

Photocaged chemical inducer (IPTG) [11] and photocaged enzyme (T7 RNA polymerase) [14] or synthetic light-sensing two-component systems [15], [16] have been used for the development of light-regulatable gene expression systems. Caged molecules (inducer or enzyme) carrying a protecting group can be removed by irradiation with UV light to restore their chemical and biological functions. The photo-induced reaction leads to an irreversible release of the caging group, and therefore, it does not allow switchable gene expression. Previously, a marker gene expressed under promoters with a GAL4 DNA-binding site was shown to be induced by red light and abrogated by subsequent far-red light in yeast cells expressing 2 chimeric proteins, namely, a phytochrome (PhyB)-GAL4 (DNA-binding-domain) fusion and a PIF3-GAL4 (activation-domain) fusion [15]. Recently, a light-sensing system was constructed in *Escherichia coli* by using a synthetic combination of a two-component regulatory system of *E. coli* (EnvZ/OmpR) and a light-sensing phytochrome from cyanobacteria (Cph1) [17]. This system could not provide rapid reversible control of gene expression or switchable control of the expression of target genes [16].

In this study, we improved the previously engineered light-regulated two-component system to perform rapid and switchable regulation of gene expression in *E. coli* and proposed possible ways to fine-tune the system for tight control. The chromosomally encoded λ repressor was produced in the absence of light (darkness), which, in turn, blocked the expression of green fluorescent protein (GFP) from a constitutive promoter on a reporter plasmid. GFP levels were monitored for different light stimuli (turn on/off or switchable light stimuli) to determine the efficiency of the system.

## Materials and Methods

### Bacterial Strains and Growth Conditions

The bacterial strains used in this study are listed in table 1. *E. coli* MG1655 was used as the parental strain. All strains were grown in Luria–Bertani (LB) medium at 37°C with rotation at 200 rpm. Strains carrying temperature-sensitive plasmids were maintained at 30°C. Media were supplemented with antibiotics at suitable concentrations (25 µg/mL kanamycin, 25 µg/mL ampicillin, and 15 µg/mL chloramphenicol).

**Table 1:** Strains and plasmids used in this study.

Strains/Plasmids	Description	Reference/source
***E. coli***		
MG1655	K-12/F-lambda-*ilvG*-*rfb*-50 *rph*-1	[27]
JM1012	MG1655/P*_ompC_::c*I-LVA Δ*envZ*	This study
JM1013	JM1012/pPL-PCB, pCph8, pJM1	This study
JM1014	JM1012/PL-PCB, pCph8, pJM2	This study
**Plasmids**		
pPL-PCB	p15a *ori.*, *pcyA, ho1* gene, Ap^R^	[22]
pCph8	ColE1 *ori.*, e*nvZ-cph1* gene, Cm^R^	[17]
pSIM5	pSC101 *ori*., *exo, bet, gam* gene, Cm^R^	[20]
pCP20	Yeast FLP recombinase gene, Ap^R^, Cm^R^	[28]
pKD13	FRT-flanked kanamycin resistance	[19]
pEDL3	pSC101* *ori.*, *lacZ*, *c*I*-*LVA, *luxI*, *luxR* gene, Ap^R^	[6]
pProbe-NT′	pBBR1 ori., KmR	[18]
pJM1	pBBR1 *ori.*, pProbe-P_L_-*gfpuv* with strong RBS, Km^R^	This study
pJM2	pBBR1 *ori.*, pProbe-P_L_-*gfpuv* with weak RBS, Km^R^	This study
pPro7(E)-*gfp*	pPro24(E)-*gfpuv* derivative with AAGAAGG RBS, Ap^R^	[9]

Ap^R^ and Cm^R^ indicate resistance to ampicillin and chloramphenicol, respectively. 2-MC, 2-methylcitrate; RBS, ribosomal binding site.

### Strain Construction

To construct JM1012, the *ompC* gene on the chromosome of the wild-type *E. coli* MG1655 was replaced with the λ *c*I gene containing a rapidly degradable LVA degradation tag (AANDENYALVA) at the C-terminus of the gene product CI protein (CI-LVA) [5], [18] and the chromosomal *envZ* gene was deleted. Primers used in this study are listed in table 2. For *envZ* gene deletion, the kanamycin cassette was amplified from pKD13 using primers 1 and 2 containing 50-bp homology extensions flanking the *envZ* gene on the chromosome. For integration of the *c*I gene with the LVA tag, the λ *c*I repressor gene was amplified from pEDL3 using primer 3 containing 50-bp homology extensions flanking the *ompC* gene and primer 4 carrying a 20-bp overhang that is homologous to primer 5. The kanamycin cassette was amplified from pKD13 using primer 5 and primer 6 carrying a 50-bp overhang that is homologous to the *ompC* gene. Two PCR product fragments were sewn together using splicing by overhang extension (SOE)-PCR [19]. Gene deletion and integration experiments were performed using the λ-Red-mediated recombination system [19], [20]. Briefly, cells harboring pSIM5 were grown to the mid-log phase, and induced by thermal inactivation of the λ CI857 repressor at 42°C for 15 min. Cells were made electrocompetent and transformed with the PCR products containing the kanamycin-resistance cassette flanked by short regions homologous to the target gene. Transformant colonies carrying the desired modification were directly selected on kanamycin-containing LB agar plates. Genomic DNA was isolated from the transformants, and the target region was PCR-amplified and sequenced to confirm site-specific modification. The kanamycin cassette was then removed using the Flp recombinase system borne on the pCP20 plasmid [19].

**Table 2:** Primers used in this study.

Plasmid construction	Primer sequence
***envZ*** ** deletion** a	
primer 1	5′−AACGGGAGGCACCTTCGCCTCCCGTTTATTTACCCTTCTTTTGTCGTGCC **TGTAGGCTGGAGCTGCTTCG**−3′
primer 2	5′−ACCGTCTGGGGTCTGGGCTACGTCTTTGTACCGGACGGCTCTAAAGCATG **ATTCCGGGGATCCGTCGACC**−3′
***c*** **I insertion** a	
primer 3	5′−TGGCATAAAAAAGCAAATAAAGGCATATAACAGAGGGTTAATAACATGAGCACAAAAAAGAAACCATTAACACAAGAG−3′
primer 4	5′−**GGTCGACGGATCCCCGGAAT**TTAAGCTACTAAAGCGTAGTTTTCGTCGTT−3′
primer 5	5′−**ATTCCGGGGATCCGTCGACC**−3′
primer 6	5′−GCAGGCCCTTTGTTCGATATCAATCGAGATTAGAACTGGTAAACCAGACC **TGTAGGCTGGAGCTGCTTCG**−3′
**P_L_-GFP[LVA tag] into pProbe NT′** b	
primer 7	5′−CGGAATTCCGTAAGCACCTGTAGGATCGTACAGGTTGACAACAAGAAAATGGTGTGTTATA**GTCGAATAACACCGTGCGTGTT**−3′
primer 8	5′−**GTCGAATAACACCGTGCGTGTT**GACTATTTTACCTCTGGCGGTGATATATAAGGAGGAAAAACATATGAGT−3′
primer 9	5′−**GTCGAATAACACCGTGCGTGTT**GACTATTTTACCTCTGGCGGTGATATATGGATTAGAAAAACATATGAGT−3′
primer 10	5′−GAATTAAGCTTCTGCAGTCGACTTAAGCTACTAAAGCGTAGTTTTCGTCGTTTGCTGCAGGCCTTTTGTATAGTTCATCCATGCCAT−3′

a In *envZ* deletion and *c*I insertion, gene specific regions are underlined and P1 and P4 regions are indicated in bold letters.

b In pJM1 and pJM2 construction, GAATTC, *Eco*RI site; GTCGAC, *Sal*I site; AGCTACTAAAGCGTAGTTTTCGTCGTTTGCTGC, LVA site; AATAACACCGTGCGTGTT, CI binding site 1; TTTACCTCTGGCGGTG, CI binding site 2; AAGGAG, strong RBS (0.3); GGATTA, weak RBS (0.1). Homologous regions of primer 7 and primer 8 are indicated in bold letters.

### Plasmid Construction

All DNA manipulations were performed using established protocols [21]. The pPL-PCB [22] and pCph8 [17] plasmids were used for expression of the light-sensing system in JM1012. To construct the pJM1 and pJM2 reporter plasmids, a promoter (P*_L_*) containing 2 λ CI-binding sites, a ribosome-binding site (RBS) (AAGGAG as a strong RBS and GGATAA as a weak RBS), and a reporter gene, *gfpuv*, were cloned into the *Eco*RI and *Sal*I sites of pProbe-NT′ [18]. Primers 7 and 8, or 9 and 10 were used to construct the pJM1 and pJM2 reporter plasmids, respectively. Plasmid pJM1 and pJM2 are different from each other in their RBS sequence, with strong RBS in the former and weak RBS in the later. RBS sequences were designed using the RBSDesigner software (http://rbs.kaist.ac.kr/index.html) that determines the strength of a particular RBS (translational efficiency) using a relative scale from 0 to 1 [23]. The translational efficiency is directly proportional to the rate of translation initiation. The translational efficiency of a popular RBS of *E. coli* (AAGGAG) used for pJM1 was predicted to be about 0.3. The weak RBS (GGATAA) used for pJM2 was designed by setting the translational efficiency to 0.1.

### Light-controllable 24-well Bioreactor

**Figure 1:**
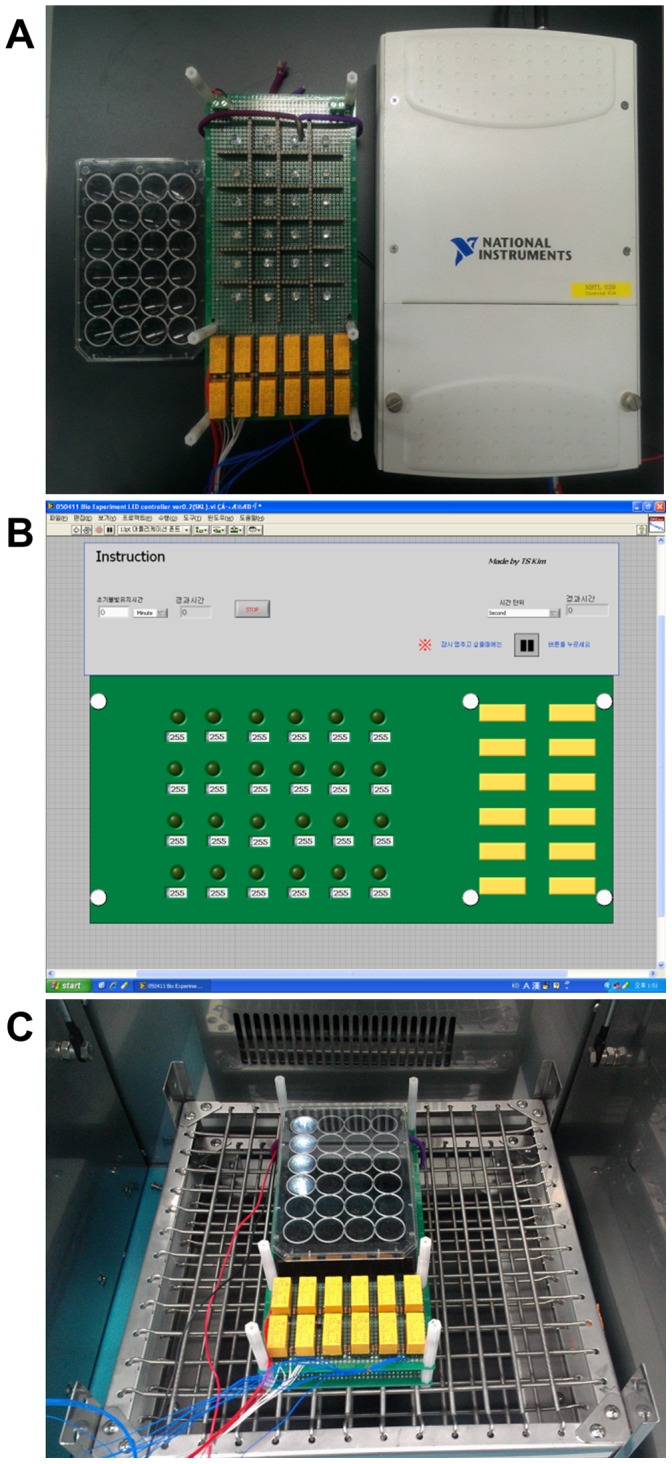
A light-controllable bioreactor system. (A) A homemade electric circuit and a data acquisition system to determine the effect of light duration on gene expression in the bioreactor was combined with a black, commercial 24-well plate. (B) The software interface is shown. Culture conditions in each well, such as the time of exposure to light, were controlled using the software LabVIEW. (C) This figure shows the bioreactor mounted on a rotating orbital shaker placed in an incubator.

A light-controllable 24-well bioreactor was built by using a commercial 24-well plate and ordinary white light-emitting diodes (LEDs) (IWL-W5R30F, Itswell, Incheon, Republic of Korea). In short, each LED was soldered on a printed circuit board and surrounded with regular black plastic walls to prevent light from interfering with neighboring wells and to tightly secure the 24-well plate to the printed circuit board. Since each LED was individually connected to the digital output of a data acquisition system (NI USB 6221; National Instruments, Austin, TX, USA) that has 24 digital output channels, it was possible to manipulate each LED duty cycle independently by using LabVIEW (Version 11.0; National Instruments, Austin, TX, USA) software (Fig. 1). Hence, the built photobioreactor made it possible to investigate the effect of light on gene expression levels in both parallel and high-throughput manners. The side walls of the 24-well plate were opaque for minimizing the effect of light reflected from neighboring wells, whereas the bottom was transparent, allowing deeper penetration of light through the cells with various duty cycles of light. Use of a timer control aided in rapid switching ON/OFF of the light uniformly across the 24 wells. For cell culture, the photobioreactor was operated in a regular shaking incubator at 150 rpm and at 37°C. The light exposure time varied from 0 h to 12 h.

### Measurement of GFP Expression

The JM1012 strain containing the 3 plasmids (pPL-PCB, pCph8, and pJM1/pJM2) was grown overnight in 5 mL of LB medium supplemented with 3 different antibiotics at 37°C and at 150 rpm. Twenty microliters of overnight-grown culture was transferred into 2 mL of fresh LB medium in each well of a light-controllable 24-well plate. After incubating cells in the microplate for 2 h, until the OD_600_ reached approximately 0.3, cells were induced by light. For induction, the cells were exposed to the light transmitted from a light-emitting diode through the bottom of the wells, using the light-controllable bioreactor. The quantity of GFP in cultures was monitored using a SpectraFluor Plus plate reader (TECAN; Durham, NC, USA) with excitation/emission at 485/535 nm at a constant gain value of 30. GFP expression in single cells was quantified using a FACSCalibur flow cytometer (BD Biosciences, Franklin Lakes, NJ, USA). Signals arising from viable cells were analyzed using FlowJo software (Tree Star Inc., Ashland, OR, USA). Before injection into the flow cytometer, the cells were induced by different durations of light (0, 1, 2, 3, 4, 5, or 6 h). As a control, cells carrying pPro7(E)-*gfp* were grown to an OD_600_ of 0.3 and induced with varying concentrations of propionate. Cells in either case were harvested after 6 h by centrifugation (1 min at 13,000×g, 4°C) and washed twice with phosphate-buffered saline (PBS). The concentration of cells was adjusted to approximately 3×10^6^ cells/mL in PBS. Approximately 1×10^5^ cells were counted for each sample.

## Results and Discussion

### Light-switchable Gene Expression System

**Figure 2:**
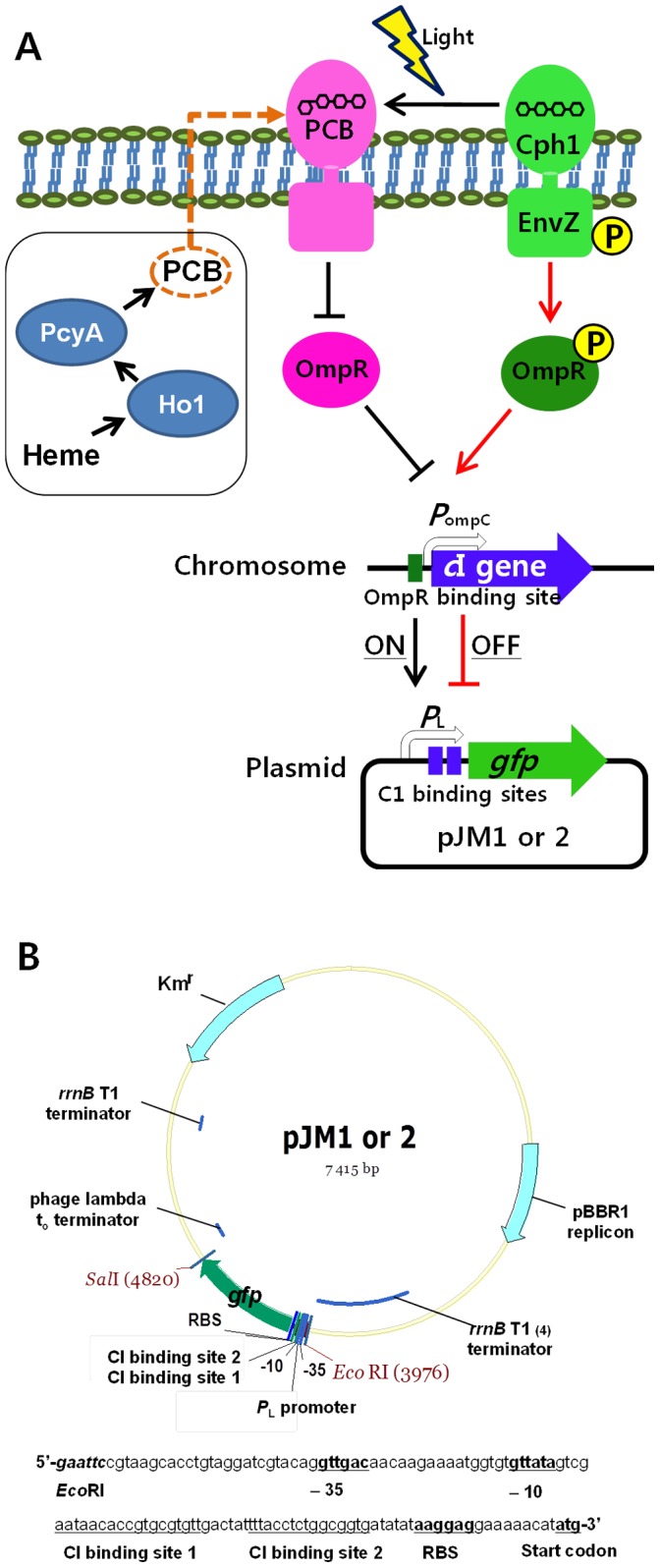
Schematic diagram representing the light-switchable gene expression system. (A) The phycocyanobilin chromophore PCB is synthesized by Ho1 and PcyA enzymes expressed from the pPL-PCB plasmid. The hybrid photoreceptor, Cph8, is a fusion of the photoreceptor Cph1 and the EnvZ histidine kinase expressed from the pCph8 plasmid. The λ *c*I gene containing a rapidly degradable LVA degradation tag is expressed under the control of the P*_ompC_* promoter. (B) The reporter plasmids, pJM1 and pJM2, have a *gfpuv* gene under the control of the P*_L_* promoter with 2 CI-binding sites and either a strong or weak RBS. EnvZ, *E. coli* osmosensor histidine protein kinase/phosphatase; OmpR, the cytoplasmic response regulator of the EnvZ/OmpR two-component system; CI, lambda repressor; T1_(4)_, 4 tandem copies of the T1 terminator from the *E*. *coli rrnB*1 operon; PCB, phycocyanobilin; Ho1, heme oxygenase; PcyA, biliverdin reductase; Cph1, red light-responsive domain of strain PCC 6803 phytochrome; RBS, ribosomal binding site.

The major components of the light-switchable gene expression system used in this study include the strain JM1012 (MG1655/P*_ompC_*::*c*I-LVA Δ*envZ*) and 3 plasmids: 2 for sensing light (pPL-PCB and pCph8) and 1 reporter plasmid (pJM1/pJM2) (Fig. 2). The pPL-PCB plasmid expressing *Synechocystis* sp. PCC 6803 heme oxygenase (Ho1) and biliverdin reductase (PcyA) genes leads to the biosynthesis of the chromophore phycocyanobilin (PCB) [22]. PCB is the major light-sensing component. The pCph8 plasmid harbors a fusion gene encoding a red light-responsive domain (Cph1) of the strain PCC 6803 phytochrome and an intracellular histidine kinase (HK) of *E. coli* EnvZ [17], [22]. The chimeric light-receptor Cph8 combines with PCB to form a holophytochrome and autophosphorylates its HK domain in darkness. In the absence of light, the chimeric light receptor transfers its phosphate group to a transcriptional activator, OmpR. Phosphorylated OmpR binds to the *ompC* promoter (P*_ompC_*) and, as a result, activates the expression of CI-LVA that is expressed under the control of the *ompC* promoter on the *E. coli* chromosome (Fig. 2A). The expressed CI-LVA, in turn, blocks the transcription of *gfp* from the promoter (P*_L_*) with 2 λ repressor operators present on the reporter plasmid (Fig. 2B). Therefore, darkness results in the production of CI-LVA and subsequent repression of GFP expression, while exposure to light relieves this repression, as the λ repressor is no longer produced. A rapidly degradable CI-LVA would favor rapid turnover of the repressor, and therefore, efficient and rapidly switchable control of gene expression [6].

### Turn-on Control of Gene Expression

**Figure 3:**
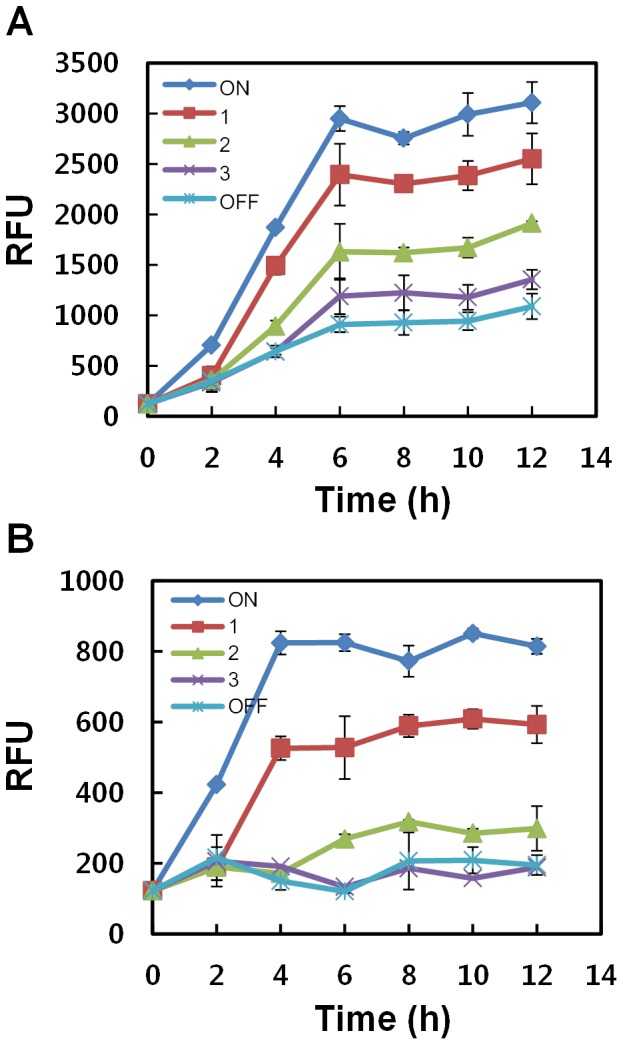
Turn-on control to switch the genes from the OFF state to the ON state. The cells harboring (A) pJM1 with a strong RBS (strain JM1013) or (B) pJM2 with a weak RBS (strain JM1014) were grown overnight in 5 mL of LB medium and subcultured (100∶1) into fresh LB medium (2 mL in the light-controllable 24-well plate). The cells were incubated in the microplate for 2 h until OD_600_ reached approximately 0.3, and then were initially maintained in darkness for 1, 2, or 3 h, and then, light stimulus was provided until the end of the experiment. Relative fluorescence per unit of OD_600_ (RFU) was measured. Data are presented as mean ± SD and basal values were not subtracted.

To determine whether this improved system could be used for temporal regulation of gene expression, the JM1013 and JM1014 strains were exposed to light at different time points following inoculation. Initially, the cells were maintained in darkness for a period of 1, 2, or 3 h, and then, light stimulus was provided (Fig. 3). GFP expression was directly proportional to the duration of the light stimuli. This expression system could provide a relatively easy way to initiate a temporal change in induction time, which, in turn, could control gene expression levels. However, a delay was observed between the time points at which the light stimulus was provided and the gene expression was detected. This delay was possibly due to the time required by the system for the inactivation of OmpR, degradation of the CI repressor, and expression/folding of GFP. According to a previous study [24], the time required for folding of GFPuv is approximately 45 min.

A significant difference was observed in GFP expression between the strong and weak RBSs. Although the strong RBS favored a higher level of GFP (Fig. 3A), leaky expression from the weak RBS (Fig. 3B) was much lower than that from the strong RBS (Fig. 3A). The basal GFP values from *E. coli* carrying the *gfp* gene under the control of weak RBS in its OFF state (Fig. 3B) was found to be similar to the background levels observed in *E. coli* harboring an empty vector (data not shown). In addition, the fold induction between the OFF and ON states was higher with a weak RBS (4.6-fold) than with a strong RBS (3-fold). This result indicates that leaky expression can be mitigated by using the weak RBS but this also results in a reduction in the total amount of protein produced. The weak RBS system present in the plasmid pJM2 would be more desirable for some metabolic engineering practices involving toxic gene products, as it provides considerably less basal expression.

When grown in LB medium, *E. coli* strains reached late-log phase approximately 3 h after cell inoculation. GFP expression in cells maintained in darkness for 3 h was slightly higher than background levels (for strong RBS, Fig. 3A) or comparable to background levels (for weak RBS, Fig. 3B). These data indicate that cells in the stationary phase may not be strongly induced by the *E. coli* EnvZ/OmpR two-component system. Taken together, our results suggest that tightly controlled expression of different proteins would be possible by coordinating many factors such as promoter strength, RBS strength, operator affinity to repressor, and regulatory protein amount.

### Turn-off Control of Gene Expression

**Figure 4:**
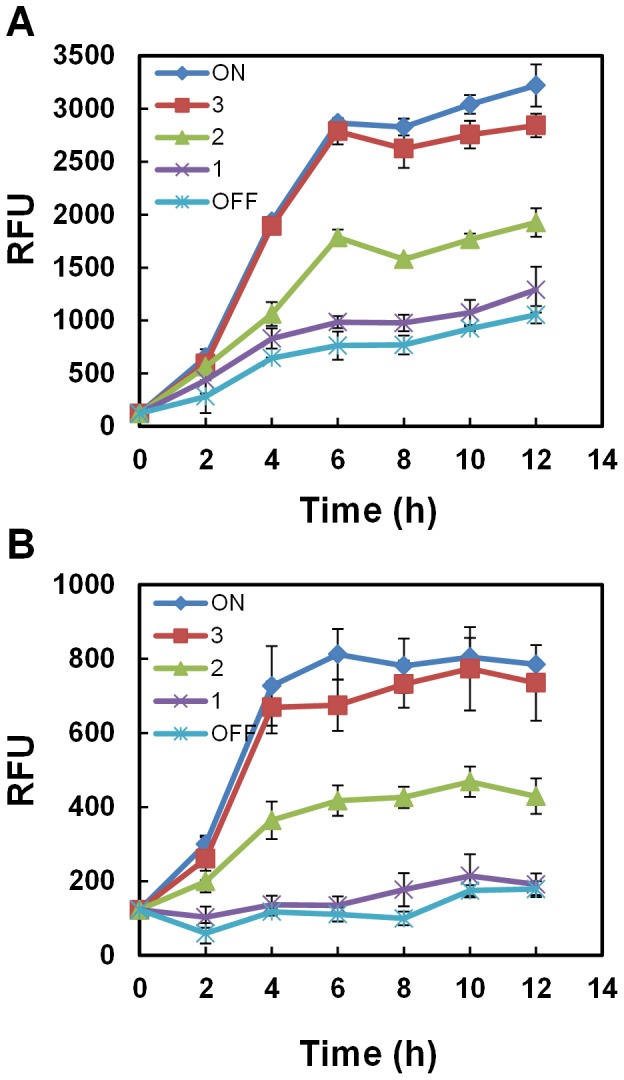
Turn-off control to switch the genes from the ON state to the OFF state. The cells harboring (A) pJM1 with a strong RBS (strain JM1013) or (B) pJM2 with a weak RBS (strain JM1014) were inoculated and incubated in the microplate for 2 h until the OD_600_ reach approximately 0.3, and then were initially exposed to light for 1, 2, or 3 h, and then, switched to darkness until the end of the experiment. RFP fluorescence per unit of OD_600_ was measured. Data are presented as mean ± SD and basal values were not subtracted. This experiment was performed using the same culture method as described in Fig. 3.

To determine whether this gene expression system could be used to turn gene expression off at a desired time, the light stimulus was withdrawn after 1, 2, or 3 h from the JM1013 and JM1014 strains growing in the presence of light. The efficiency with which gene expression was turned off was determined by analyzing the GFP expression levels (Fig. 4). Our results indicate that a shift from conditions of light to darkness blocked the apparent increase in GFP expression within a short time of removal of the light stimulus. The short delay to switch gene expression off can possibly be attributed to the time the system requires to phosphorylate OmpR and produce CI-LVA repressors. It seems, therefore, that reversal from light to dark conditions in the *E. coli* cells effectively terminates gene expression, presumably by phosphorylating OmpR in darkness, which binds to the *ompC* promoter and activates expression of the CI-LVA repressor, which, in turn, represses the λ repressor-repressible promoter (P*_L_*) on the reporter plasmids.

Exposing the cells to light for 3 h from the time of inoculation was sufficient to achieve net GFP expression similar to that achieved from 12 h of exposure to light. This could be because the cells growing in LB medium may enter a stationary phase after 3 h, wherein protein expression may not be active. Consistent with this, providing light stimuli after 3 h of inoculation did not significantly induce GFP expression (Fig. 4A&B). Considering these data together, it could be concluded that, with the light switchable system, maximal induction can be achieved by inducing cells in log phase and that the relative expression level is reduced when cells are induced in the stationary phase.

Similar to chemical-inducible systems, where concentrations of the inducer are directly proportional to levels of gene expression, this light-inducible system can also control the amount of a recombinant protein depending on the duration of exposure to light. Unlike the chemically inducible gene expression systems that cannot switch gene expression off after induction in a batch culture, this system has the advantage of turning gene expression off at any given time point after induction, even in a batch process.

### Measurement of Gene Expression at the Single Cell Level

**Figure 5:**
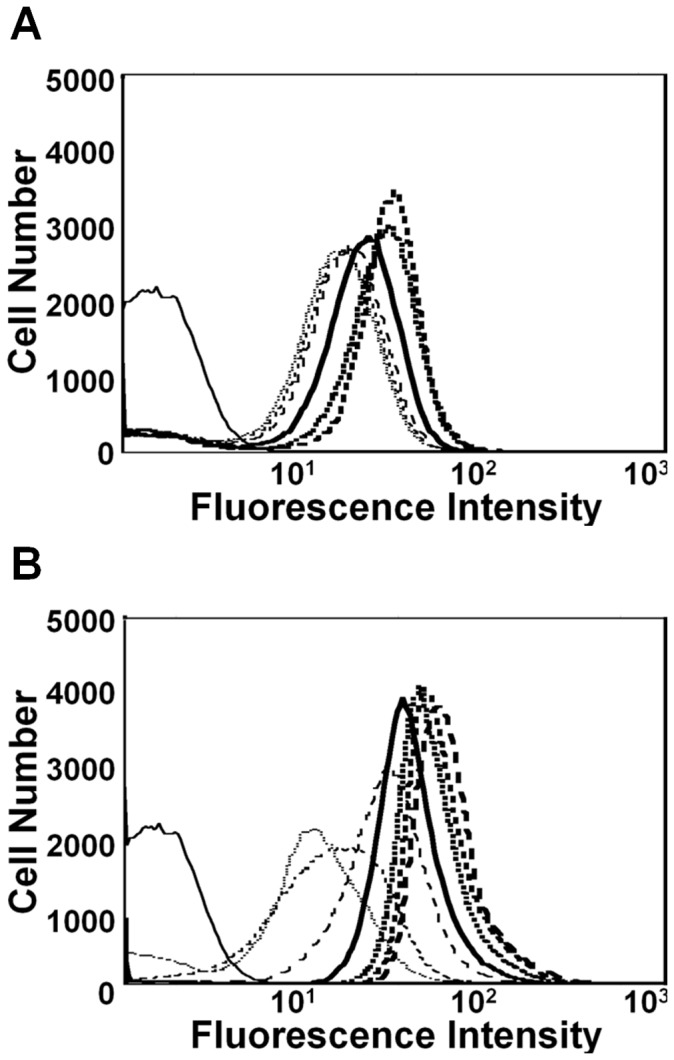
Histograms showing the number of cells with a given fluorescence in *E. coli* MG1655 cultures harboring pPro7(E)-*gfp* and JM1013. Cultures of (A) the MG1655 harboring pPro7(E)-*gfp* and (B) the JM1012 harboring the 3 plasmids (pPL-PCB, pCph8, and pJM1/pJM2) were analyzed. *E. coli* harboring pET28a empty vector was used as a control. Cell cultivation was performed as described in Materials and Methods. The fluorescence in single cells was measured after induction of 6 h with different propionate concentrations or light exposure times. Propionate concentrations: control (solid line), 0 mM (dot line), 0.2 mM (dashed line), 0.8 mM (long-dashed line), 3.2 mM (bold solid line), 12.6 mM (bold dotted line), and 50 mM (bold dashed line). Light exposure times: control (solid line), 0 h (dotted line), 1 h (dashed line), 2 h (long-dashed line), 3 h (bold solid line), 4 h (bold dotted line), 5 h (bold dashed line), and 6 h (bold long-dashed line).

For many functional genomic and metabolic applications, it is necessary to have a synchronized behavior in a population of cells. Flow cytometry was used to assess whether a homogenous shift in gene expression could be achieved using the light-switchable expression system by varying the duration of illumination. As shown in figure 5B, when induced with a particular light exposure time, the entire cell population exhibited similar GFP levels which increased in a uniform fashion with the increase in the duration of light exposure time, indicating that there are essentially no unresponsive cells across the population. The use of a photobioreactor could allow efficient penetration of light through dense culture broth and elicit homogenous induction.

A chemically inducible *prpR*-P*_prpB_* expression (pPro) system was used as a control, since the system was previously reported to exhibit homogenous GFP expression at the single cell level. From figure 5, it is clear that peak width of the light-inducible system is narrower than that of the pPro system. In addition, the light-switchable gene expression system was highly regulatable compared with the pPro system with maximal 1,500-fold induction in that it showed lower basal expression and higher induced expression than the pPro system [9], [25]. Since the strength of the pPro system has been shown to be comparable to that of other strong expression systems, such as the P*_trc_* promoter system, this light-switchable system may also be effective in overexpression of target proteins. It can be concluded that gene expression in the JM1013 strain occurs in a strictly concerted manner rather than in the all-or-none manner observed in most popular promoter systems, such as the *araBAD* and *lac* promoters [25], [26]. These findings suggest that this system could be a very attractive option for researchers in systems biology, metabolic engineering, and synthetic biology, where the precise and temporal control of gene activation is required.

### Switchable Control of Gene Expression

**Figure 6:**
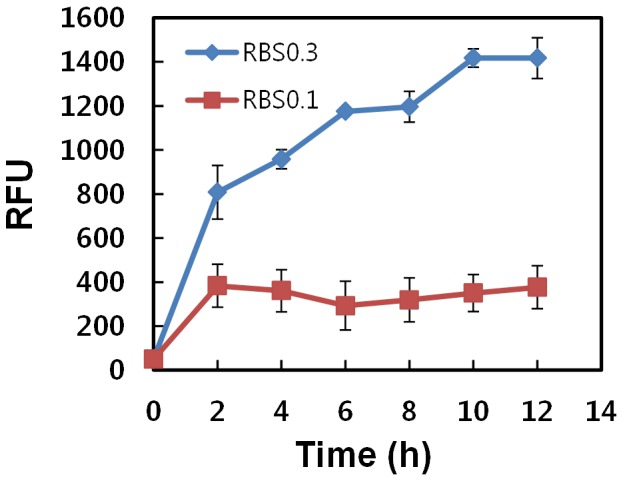
Switchable control of GFP expression. Graph shows variation in GFP expression with respect to an oscillating light source (light/dark cycle at 2-h intervals) from JM1013 (diamonds) and JM1014 (squares) strains. Data are presented as mean ± SD and basal values were not subtracted. This experiment was performed using the same culture method as described in Fig. 3. RBS0.3, strong RBS; RBS0.1, weak RBS.

To verify whether light can be used to achieve reversible switching ON or OFF of gene expression, cells were exposed to a repetitive light-dark cycle every 0.5, 1, or 2 h. Because the λ CI repressor carries a degradation tag, it can be easily degraded, which results in stoppage of GFP expression when the light stimulus is absent. As expected, darkness resulted in an almost complete halt in GFP expression. Figure 6 clearly indicates that it is possible to switch cells from the ON to the OFF state rapidly. Continuous oscillation of light stimuli every 2 h showed a stepwise increase in GFP in the JM1013 strain with a clear distinction between GFP levels in the ON versus OFF state (Fig. 6). Recently, Tabor *et al*. used a combination of green and red light sensitive two-component systems and CI repressor to demonstrate spatial control of the expression of multiple gene by employing two different wavelengths of light [16]. However, they did not demonstrate rapid reversible control of the expression of target genes [16], probably because of long time periods required to switch between ON an OFF states. Unlike the previous system, the presence of a single copy of rapidly degradable CI-LVA on the chromosome in our system may be an efficient way to reach a steady state of expression within shorter duration [16]. Hence, we were able to see a clear reversion between the ON and OFF states of the target gene, resulting in efficient switching.

Interestingly, the strain with weak RBS, JM1014, did not exhibit light-switchable gene expression, indicating that the RBS was too weak to distinguish precisely between the ON and OFF states. From this finding, it is clear that optimizing the RBS strength of the target gene might also be an important factor to achieve rapid switchable regulation. The strain with strong RBS, JM1013, showed a stepwise increase rather than the expected sinusoidal oscillation. This could be because GFP is a relatively stable protein, which cannot be degraded within 2 h. Cells containing GFP have been reported to show no decrease in fluorescence over at least 24 h [5]. In order to achieve an efficient sinusoidal oscillation, it is very important to know how fast the system responds to alteration of illumination. To date, however, no study has shown sinusoidal oscillatory control of gene expression in *E. coli* in a batch process. We tested our system using a rapidly degradable GFP-LVA [18] that has a half-life of approximately 40 min to show a sinusoidal oscillation in gene expression. However, the rate of GFP degradation was faster than that of GFP production, and hence, GFP expression was too low to be detected (data not shown). These findings suggest that switchable gene expression needs to be tightly regulated by several factors, such as mRNA stability, target protein stability, regulatory protein levels, and binding affinity between the regulatory protein and its binding site.

### Conclusions

Since light as an inducer of gene expression has several advantages over chemical inducers, light-switchable regulation of gene expression has potential benefits across diverse fields in science and engineering. Light-switchable gene expression system and photobioreactor were designed and constructed to achieve rapidly reversible and tightly regulated expression of the target gene. The light-switchable system provides a dose-dependent reversible control of gene expression that was homogenous across an entire population. The photobioreactor is an easy-to-use device that simplifies the task of fine control of duration of light. Therefore, this system could be effectively applied to metabolic engineering and synthetic biology in order to achieve switchable expression of either chromosomal or plasmid-borne genes.
